# How longitudinal data can contribute to our understanding of host genetic effects on the gut microbiome

**DOI:** 10.1080/19490976.2023.2178797

**Published:** 2023-02-16

**Authors:** Laura Grieneisen, Ran Blekhman, Elizabeth Archie

**Affiliations:** a Department of Biology, University of British Columbia, Okanagan Campus, Kelowna, BC, Canada; b Section of Genetic Medicine, Department of Medicine, University of Chicago, Chicago, IL, USA; c Department of Biological Sciences, University of Notre Dame, Notre Dame, IN, USA

**Keywords:** Gut microbiome, host genetics, time series, host-microbiome interactions, heritability, plasticity

## Abstract

A key component of microbiome research is understanding the role of host genetic influence on gut microbial composition. However, it can be difficult to link host genetics with gut microbial composition because host genetic similarity and environmental similarity are often correlated. Longitudinal microbiome data can supplement our understanding of the relative role of genetic processes in the microbiome. These data can reveal environmentally contingent host genetic effects, both in terms of controlling for environmental differences and in comparing how genetic effects differ by environment. Here, we explore four research areas where longitudinal data could lend new insights into host genetic effects on the microbiome: microbial heritability, microbial plasticity, microbial stability, and host and microbiome population genetics. We conclude with a discussion of methodological considerations for future studies.

## Plain Language Summary

For humans and animals, host genes play a role in shaping the gut microbiome. However, measuring these effects is difficult because host genetic and environmental similarities are often correlated. For instance, relatives often live together and share similar diets and lifestyles—forces that can also change gut microbial communities. Watching the microbiome over time, through longitudinal sampling, can help solve this problem by breaking gene-environment correlations. Here, we review this idea and several other research areas where longitudinal data will be helpful for understanding host genetic effects on the microbiome. We believe this approach will shed new light on the evolution of host–microbe relationships and can inform new microbiome therapies.

## Introduction

Gut microbial taxa – including those under host genetic influence – have widespread effects on host health and physical functioning, as reviewed in recent studies.^1–3^ They help to digest food,^4,5^ influence host behavior,^6,7^ and regulate host gene expression,^8,9^ metabolites,^10,11^ and immune mechanisms.^12^ On evolutionary scales, relationships between gut microbes and host genes may facilitate local adaptation to novel environments in humans (as reviewed in ^2^), shape adaptive phenotypic plasticity across mammalian hosts (as reviewed in ^13^), affect host genetic divergence between populations,^14^ and even, some argue, lead to selection of a host and its microbes as a single evolutionary unit.^15^ Given these evolutionary implications, it may seem surprising that only a minority of microbiome taxa are associated with host genetic variation. For instance, few genetic variants in hosts are consistently identified as having gut microbial correlations across population-specific studies (reviewed in ^3^). Further, in humans, very few microbial taxa are heritable (an estimated 3–13%, on average; ^16–19^), and those that are most frequently heritable have widely varying heritability estimates across studies (e.g., Christensenellaceae, one of the most consistently heritable taxa in humans, exhibits heritability ranging from 0.31 to 0.64 ^20^).

A major challenge with estimating microbiome heritability is the difficulty in isolating host genetic effects from other drivers of microbiome variation–especially variation in the environment. Host environments play a strong role in shaping gut microbiome composition,^21,22^ and genetic similarity between hosts is often confounded by shared environments (e.g., diet and lifestyle ^23^). As such, even studies with large sample sizes struggle to detect host genetic effects in the face of environmental correlations.^3^ For example, Gacesa et al. ^24^ found that across 8,208 individuals from 2,756 family units, only 6.6% of microbes were heritable. In contrast, 48.6% of microbial abundances were affected by cohabitation, but because relatives often cohabitate, the correlation between shared genes and shared housing may have obscured the researchers’ ability to detect heritable microbes.

This complication is exacerbated by the dynamic nature of microbiome community composition within a host over time. For example, a study in which 20 individual hosts were sampled daily for 6 weeks found that variations in the abundances of most microbes were greater within individual hosts than between hosts.^25^ An additional complication is that genetic effects on dynamic host traits can be themselves dynamic. For example, the heritability of dynamic host traits, such as body mass index (BMI), changes over an individual’s lifetime as the relative importance of environmental and behavioral factors increase compared to genetic effects.^26,27^ Similar age-related changes in heritability have also been found in the gut microbiome.^21^

To date, most research on host genetic effects on the gut microbiome relies on cross-sectional data, but time-series data can supplement our understanding of the relative role of genetic processes in the microbiome in ways that cross-sectional data cannot. Broadly, sampling the same individual over time can help to reveal environmentally contingent host genetic effects, both in terms of controlling for environmental differences and in comparing how genetic effects differ by environment. Longitudinal sampling can also minimize cohort effects. For example, ^20^ note that in a study of age and microbiome composition, the association of Christensenellaceae abundance with age is based on one data point per host, such that if individuals in a certain age class have a similar diet or share other life history traits, the Christensenellaceae result may reflect between-cohort differences rather than a true age-related increase in Christensenellaceae abundance. Sampling the same host multiple times can reveal age-related changes in microbial abundances within individual hosts. Repeated sampling may also help to ameliorate the sample size and power limitations discussed in the past work^3^.

In this review, we highlight four areas where time-series data are particularly valuable for understanding host genetic effects on the microbiome and the implications of these patterns for evolution and host health. These four areas are (1) heritability of microbial phenotypes, (2) local adaptation and phenotypic plasticity of the microbiome, (3) lifetime stability and dynamism of microbial communities, and (4) microbiome population genetics. Additionally, we provide a roadmap for other researchers by summarizing methods for visualizing and modeling longitudinal microbiome data with associated host genetic information and proposing future directions for the field.

## How time series data can inform host genetics effects on the gut microbiome

### Microbiome phenotype heritability

Quantifying the heritability of complex, dynamic phenotypes requires parsing environmental effects from genetic effects. In the case of the gut microbiome, it is common to define phenotype heritability as the proportion of variation in either microbiome community composition or the abundances of individual microbial taxa that can be explained by host genetic variance.^4,21,24^ Larger sample sizes, which are often advocated for in studies of host genetic effects on microbiome composition,^24^ cannot necessarily overcome the limitation that host genetic similarity is often correlated with environmental similarity.

**Figure 1.**
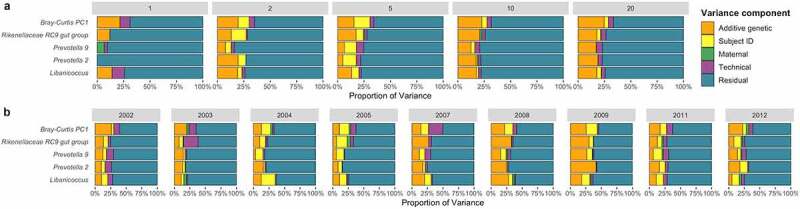
Genetic and non-genetic variance components of the microbiome for four highly heritable taxa and one metric of overall microbiome composition from up to 585 baboon subjects measured (A) at one, two, five, ten, and twenty timepoints per subject; and (B) with timepoints grouped by hydrological year, with varying numbers of timepoints per subject across a mean of 220 subjects per year. These data were generated from a data set published as part of.^21^ Figure 1A emphasizes that increasing the number of samples per subject affects our ability to detect host genetic effects, while Figure 1B shows that the relative contribution of genetic and environmental components to overall microbiome variance can change over time.

One approach that can help disentangle gene/environment correlations is to use study designs that leverage longitudinal microbiome samples from many individual hosts. For instance, a study using 16,234 samples from 585 wild baboons found that using multiple samples per host led to a striking increase in the number of heritable microbial taxa detected versus using one sample per host.^21^ Likewise, including multiple samples per subject over extended time scales can also help break the genetic relatedness/environmental similarity correlation that can affect heritability estimates in cross-sectional designs, as relatives may share environments at a given time point, but undergo individualized changes in their physical and social environments over time. Further, repeated sampling from the same individual removes cohort effects when testing how environments or host traits affect heritability estimates. For example, the relationship between host age and microbiome heritability can be better teased out when, for instance, all four-year-old hosts in the data set were not born in the same calendar year, and thus have dissimilar early life environmental effects. Comparing heritability estimates across environments is particularly important because heritability estimates of individual microbes are environmentally dependent.^1^ This effect has been demonstrated experimentally in a study of the corn rhizosphere across field sites ^28^ and in the observational study of baboon gut microbes described above.^21^ By repeated sampling of individual hosts across changing environments, it is possible to paint a broader picture of the context of the changing relative contributions of additive genetic versus environmental effects on microbial composition (as shown in Figure 1). Similar to sampling across multiple physical environments, sampling a host at multiple daily timepoints could provide an interesting contribution to our understanding of heritability, as several gut microbial taxa demonstrate a marked circadian rhythm in their relative abundances ^29^ (reviewed in ^30^).

Longitudinal microbiome data are already being used to understand heritability in some health and agricultural applications. For example, questions about the heritability of early life gut colonization and community assembly in humans have been addressed by repeated sampling of multiple sets of triplet infants.^31^ In agricultural and livestock research, understanding the relationship between host genotype, microbes, and phenotype allows for targeted breeding of desired traits,^1^ such that parsing host gene-by-environment interactions on host phenotypes, as well as gene-environment-microbiome interactions, is a high priority.^32^ One such example is a longitudinal study of piglets that determined heritable microbes are associated with growth rates.^33^ Agricultural and livestock systems also provide great experimental setups for quantifying microbial heritability in changing environmental contexts. For example, planting inbred maize lines in different fields over 2 years revealed host genotype effects on microbiome rhizosphere colonization throughout differing environments.^34^

### Phenotypic plasticity of the gut microbiome

Gut microbiome community composition is a plastic phenotype, but the dynamics of this plasticity are not well understood.^35,36^ Longitudinal data can reveal if gut microbial dynamics are individualized. Combining this information with data on host genetics could further reveal if host genotype predicts plasticity in the microbiome phenotype. For example, past studies have shown that when hosts undergo a change in their environment by moving to a new area, their microbiomes likewise change as they pick up local microbes.^37–39^ Further, the rate of local microbial acquisition varies between hosts. Six humans sampled pre-immigration and monthly for up to 9 months post-immigration to the US varied in the length of time; it took them to acquire local microbes, but by 9 months their guts had the same dominant taxa as long-term US residents.^37^ Similarly, a study of dispersing baboons found that males who had lived in a social group for less than a year varied considerably in how similar their gut microbiota were to other group members, but this variation decreased over time.^39^ Time-series samples from dispersing primates and other animals that periodically change environments, such as migratory birds, can provide natural experiments to test which environmental characteristics predict if hosts acquire local microbes, the length of time over which such shifts happen, and if the rate of acquisition has a genetic component. Quantifying the rate of change also provides insight into the mechanisms by which hosts acquire local microbes. An immediate change could suggest diet is the primary driver, whereas gradual acquisition of local microbes implicates horizontal transmission from conspecifics or other drivers.

Longitudinal studies could also further our understanding of when a plastic microbiome is adaptive and to what degree host genetics affects microbial plasticity. The ability to acquire the local microbiome may provide key fitness advantages to the host; indeed, studies have shown that bean bugs acquire bacteria from the soil that makes them resistant to pesticides,^40^ and woodrats acquire microbes that allow them to degrade dietary plant toxins.^41^ Longitudinal studies on these and other systems could further test local adaptation by experimentally swapping hosts between different environments and testing if fitness improves over time as they acquire the microbes of their new environment. More broadly, longitudinal data could reveal if host-associated microbes shift the mean or variance of a host phenotype. A shift in the mean could lead to a host phenotype better adapted to the current environment, but with less flexibility to adapt to a changing future environment. In contrast, a microbiome that increased variance in a phenotype could lead to increased capacity to adapt to environmental change.^42^

An additional aspect of microbial plasticity that could be considered in longitudinal designs is the plasticity of individual microbial taxa. Changes in microbial gene expression under different environmental conditions have been well studied in pathogenic bacteria (e.g. *Salmonella typhi* alters virulence factor expression in response to temperature ^43^), and temperature-dependent and other environmentally dependent changes in gut microbial gene expression could be a key component of temporal variation in host–microbiome interactions.^44^

The capacity to adapt to the local environment could start at birth; if there is selection for imperfect vertical transmission of microbes, it could lead to greater microbial variation between offspring, as proposed by.^45^ Microbial variation between offspring could increase fitness in variable environments, thus increasing the chances that one offspring will be adapted to the environment.^45^ These ideas could be tested by comparing temporal microbial trajectories between, for example, nestlings from similar hatching dates or pups from the same litter. Finally, longitudinal studies have the potential to provide insight into which taxa fluctuate and which ones are maintained at more constant levels, illuminating the tradeoff between plasticity and stability.

### Lifetime stability of gut microbiome phenotypes

**Figure 2.**
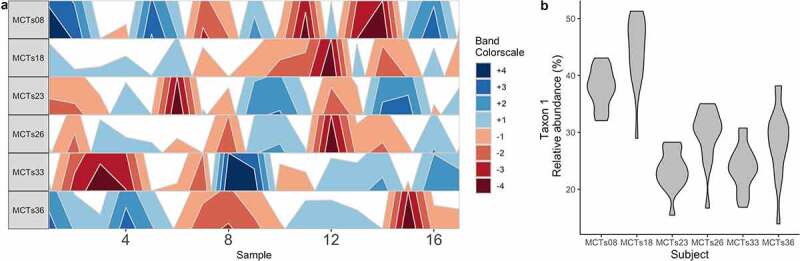
Stability and reproducibility of the microbiome. (A) A horizon plot shows how a taxon’s abundance does not change in consistent ways across time points between individual subjects. Band colors represent quartiles relative to the median. (B) A violin plot depicts the mean ± SD relative abundance of a single taxon in each individual subject, highlighting that stability is personalized. These data are from the demonstration data set from the BiomeHorizon package.^47^

Measuring microbiome stability, or the degree of microbiome change within a host over time, requires time-series data by definition. Although individual host signatures on overall microbiome community composition can be detected in samples collected years apart,^46^ individual taxa components of the microbiome are temporally dynamic, with most microbes demonstrating large fluctuations over days or weeks (as shown in Figure 2).^25^ There are two main ways that host genetic information can inform our understanding of microbiome stability. First, microbial stability itself could be under host genetic influence; i.e., the magnitude of change in individual microbial taxa over time could be quantified as a phenotype, and heritability could be estimated. Second, incorporating host genetic information into time-series models of microbial stability can provide insight into the mechanisms regulating microbial stability.

It is possible that the stability of some microbiome phenotypes has a host genetic component; i.e., stability could be heritable. In other words, host genetics could control the day-to-day change in the microbiome. Although, to the best of our knowledge, this hypothesis has not been tested with real-world data, there are several reasons why maintaining a more stable microbiome can be beneficial for the host. For example, temporal stability of ecosystem functions makes ecosystem services likewise more stable, and therefore reliable;^48^ a stable microbiome has greater resistance against colonization by invading microbes, including pathogens;^49^ and major shifts in human microbiome composition are associated with illness.^50^ However, the microbiome can also settle into an unhealthy stable state, which may contribute to chronic diseases (e.g., insulin resistance and recurrent *Clostridium dificile* infections) with resistance to medical interventions.^49^ Longitudinal data sets could be used to test if taxa that demonstrate stability over a host’s lifetime are those with abundances that are already linked to host genetics, i.e., heritable. In support of this, microbiome taxa that have abundances that are highly personalized to individual hosts tend to be more heritable than taxa that exhibit similar abundance patterns across hosts.^51^ Individual taxon stability could also be measured as genetic stability; ^46^ studied SNP (single nucleotide polymorphism) haplotypes of microbes and SVs (genomic structural variants) and found smaller differences within than between hosts and that the degree of microbial genetic stability differed between microbes. A recent study^3^ states that at least one group of bacterial species that are genetically stable over time, *Bifidobacterium*, also has high heritability. Additionally, there may be selection at the level of gene functions or pathways, rather than at the level of individual microbial taxa, and selection could also occur on pathways or functional roles filled by multiple taxa.^52^ In support of the idea that hosts and their microbiomes experience selection for some functions to be stable over time, there is a phylogenetic signature for heritability such that taxa that are more closely related–and therefore may have similar functions and occupy similar niches – may exhibit similar host genetic effects.^1^ Future studies could further explore the link between host genetics and functional stability.

Combining host genetic information with microbiome time-series data and associated environmental metadata can reveal under what circumstances microbes are stable and provide insight into stability mechanisms. Even highly heritable microbial taxa demonstrate considerable variation in abundance over a host’s lifetime,^21^ and studies have found that the stability of microbiome community composition changes with age, sickness, and other traits.^49,53^ How individual hosts vary in their degree of microbial stability, which microbes are most affected, and quantifying the degree of environmental versus genetic drivers of this variation at different temporal time scales are key questions for understanding host–microbiome interactions.^54^ A likely mechanism for host genetic control of microbial stability is changes in host gene expression, as host gene expression differences have been linked to host genetic control of the microbiome.^55^ Future work could explore if the same changes in gene expression that control microbial taxa abundances at individual time points also regulate the stability of taxa or overall community composition.

Additionally, microbiome time-series data sets that include hormone information, such as those that measure metabolites of steroid hormones in fecal samples,^56,57^ will be particularly valuable for parsing out a second potential mechanism of lifetime microbial stability. Glucocorticoids have a pervasive genetic component,^58–60^ and a study on primate microbial endocrinology^61^ proposes that host–microbiome signaling via the endocrine system may promote microbiome stability. This suggests that one potential mechanism for the host genetic influence on microbial stability is via glucocorticoid levels.

### Host and microbe population genetics

Genetic differences between host populations are also often correlated with environmental, social, and spatial characteristics, making it difficult to determine the relative contributions of these factors in shaping local microbiomes.^62^ Further, due to rapid generation times in bacteria, ecological and evolutionary processes operate at a similar temporal scale and may interact to shape microbial populations.^63^ Longitudinal data may be useful in teasing apart host population-level drivers of microbial differences by breaking these correlations and to address several unique questions: Is it possible to detect evidence of phylosymbiosis (i.e., when microbial community relationships parallel host species’ evolutionary relationships ^64,65^) or patterns of co-diversification (i.e., when the taxonomy of specific microbes parallels that of the hosts ^66^) after controlling for ecological differences? Do host populations demonstrate microbial change at different rates, and how is this rate affected by local landscape characteristics? Similarly, does a population always look like itself, or do environmental shifts cause host-associated microbial communities from temporally and geographically separated host populations to resemble each other?

Time-series data can help place evidence of phylosymbiosis or co-diversification in an ecological context. Past work has shown that the genetic structure of hosts may parallel (or not) the genetic structure of members of their microbial communities. For example, genetically similar stickleback fish populations have more similar microbes,^67^ but in humans, few microbes show biogeographical patterns that match host biogeography (reviewed in ^63^). Time-series data are useful because even among microbes whose phylogenies parallel host genetic relationships, environmental traits may modify these signatures. For example, microbiota from oysters in environmentally disturbed sites no longer exhibit genetic relationships that parallel their hosts’ population genetic structure.^68^ This phenomenon has also been shown experimentally; the microbiotas of woodrats brought into captivity and placed on uniform diets showed stronger host phylogenetic structuring than when they were in the wild, suggesting that signatures of phylosymbiosis on microbial composition may be obscured by strong environmental effects in wild populations.^69^ Time-series data can also reveal if landscape modification is reflected in microbiome changes. A recent study by Couch et al.^62^ advocates incorporating spatial elements to model how the landscape interacts with host ecology to influence population-level microbial variation. Past work has shown that geographic and environmental traits structure microbiomes between host populations of mice, tortoises, and baboons.^70–72^ By sampling host populations over time, as they shift their landscape use, it will be possible to test if changes in home range use parallel changes in microbial communities. Observational studies of long-term wild study systems will be of particular importance in answering these questions, as microbial patterns observed in controlled lab systems (or even captive animals) do not necessarily transfer to what is observed in their wild counterparts.^1,73^ These questions are also timely, as climate change causes shifts in local environments and in animal ranges.

## Methodological considerations: incorporating host genetics into longitudinal microbiome statistics

Incorporating host genetic information into longitudinal microbiome models presents a unique statistical challenge, as the wide range of longitudinal study designs that can test different aspects of host genetic effects (e.g., twin studies, family-based designs, case–control, and prospective cohort studies) have a similarly wide range of statistical approaches. Even within the microbiome-diet subfield, there are no standardized best practices for longitudinal study designs.^74^ A second complication is that some studies are constrained to irregular sampling intervals, yielding datasets with uneven time between samples. Further, the unit of analysis also varies across studies; in this review, we have focused on repeated sampling of the same individual host, but some longitudinal studies may use cage, inbred line, social group, or population as their level of analysis.

An additional set of complications that especially affect longitudinal microbiome studies are those associated with data collection and processing, which broadly fall under the issue of reproducibility in microbiome studies.^75–77^ Some common methods of sample preservation do not yield consistent microbiome profiles when subjected to freeze-thaw cycles and other stresses common to fieldwork,^75,76^ although this can somewhat be ameliorated by treating data as compositional.^78^ Batch effects must also be controlled for, as samples collected over extended time periods are often not processed at the same time.^21,79–81^ Approaches to account for batch effects include running technical replicates across multiple plates and including sequencing plates in statistical models,^21^ and using decontam ^82^ or other software to identify and remove contaminants between batches.^81^

Once sequence data are processed, the first step in any longitudinal analysis, whether it includes host genetic information or not, is visualizing which taxa are changing over time. Stream or line graphs are a common approach for visualizing temporal microbial changes for a handful of microbes in a limited number of hosts.^83–86^ As an alternative, horizon plots can show the temporal dynamics of more taxa in a more condensed visual space. Several R packages provide horizon plot functionality, including CNEr and TSFEL.^87,88^ BiomeHorizon is the first R package designed to apply horizon plots to microbiome data.^47^

There are several other software packages in R and other statistical environments that are specifically designed to analyze longitudinal microbiome data, and future studies could explore integrating host genetic information as well. TIME, a web-based interface, guides users through multiple workflows to visualize microbial abundances and co-occurrences over time, and to predict causality (dynamic time warping, Granger causality, and Dickey–Fuller tests)^86^. Ridenhour et al. propose an autoregressive integrated moving average (ARIMA) time-series model modified to handle microbiome dynamics^89^. This model uses untransformed count data as the input, and incorporates environmental differences. A study by Wanger et al.^90^ focus on time-varying analyses of overall changes in microbial diversity. Chen and Li^91^^ʹ^proposed ZIBR (two-part zero-inflated beta regression model with random effects) model accounts for the high number of zeros in longitudinal data. SynTracker is designed for the level of microbial strains^92,93^ and provides a user-friendly walkthrough of microbiome time-series analyses, as well as R and matlab tutorials. For a more detailed discussion of statistical challenges and approaches in analyzing longitudinal microbiome data, see the thorough review on the topic by Kodikara et al.^94^

Host relatedness data may also be integrated into other common models for measuring temporal variation in ecological systems, including generalized Lotka–Volterra equations,^95^ multispecies time-series data using first-order multivariate autoregressive (MAR(1)) models ^96^), generalized additive models (GAMs),^51^ and the stochastic logistic model with environmental noise (SLM).^97^

To incorporate longitudinal data into heritability models, the Animal Model includes options for multiple samples per individual.^21,98,99^ The ACE model, which is often used in twin studies, can be modified for repeated measures.^100^ Quantitative genetics models can also be extended to include genetic effects on the host and genetics effects on the microbiome.^42^ Bayesian models could also be used, including R packages for calculating heritability estimates from generalized linear mixed-effects models using a Markov chain Monte Carlo approach (MCMCglmm and QCglmm; ^101,102^). To address the issue of compositionality that is inherent to microbiome studies and has the potential to affect heritability estimates, we recommend permuting host identity in any heritability models (e.g., Fig. S10 in a study by Grieneisen et al.^21^).

## Conclusion

The incorporation of host genetic data into longitudinal microbiome studies opens up many exciting directions in the microbiome field. The future work will leverage a combination of existing collections, natural history studies of human and wild animal populations, and controlled experimental approaches.^103–105^ Further, technical advances will improve our ability to characterize temporal changes in host gene–microbiome interactions. For example, database improvements with more GWAS studies will allow for more functional gene annotations.^63^ Although this review focused on the gut microbiome, incorporation of host genetic effects into longitudinal studies of other bodysite microbiomes, such as the oral microbiome,^106,107^ skin,^108,109^ and vagina,^110^ can provide similarly exciting insights into links between microbial dynamics and host health.

In addition to the four topic areas we focused on in this review, several recent articles highlight crucial areas where longitudinal microbiome data can contribute.^63,104,111–113^ We point out that many of these areas also have host genetic components. These include quantifying disease risk;^113^ understanding intra-individual variation;^112^ linking early life effects with lifetime microbiome consequences;^104,111^ matching microbial and host aging, modeling social dynamics and transmission, uncovering which microbial taxa are heritable, and linking microbial dynamics to host fitness;^104^ and modeling gene recombination rates and characteristics of microbes under selection.^63^

The results from such studies have broad real-world applications to human health. Detailing the relationship between the gut microbiome and host genetics could be important for translational medicine applications ^114^ and personalized therapeutics.^103^ Understanding temporal dynamics and host genotype effects could help contribute to our understanding of the stable colonization of therapeutic microbes.^63^ Further, they could contribute to the expanding field of microbiome breeding, defined as conducting artificial selection on microbiomes that “seeks to change the genetic composition of microbiomes in order to benefit plant or animal hosts”^115^.
